# A Practical Inquiry into the Causes and Frequent Failure of the Operations of Depression, and of the Extraction of the Cataract, as Usually Performed, with the Description of a Series of New and Improved Operations, &c. &c.

**Published:** 1819-07-01

**Authors:** 


*A Practical Inquiry into the Causes and frequent Failure of the Operations of Depression, and of the Extraction of the Cataract, as usually performed, with the Description of a Series of new and improved Operations, &c. &c.* By Sir William Adams, Oculist Extraordinary to the Prince Regent, &c. One vol. Octavo, pp. 414. London, 1817.

Of all the senses, by means of which we become acquainted with the material world around us, that of sight conduces most to our pleasure and to our advantage. Its range is, comparatively with the other senses, almost interminable, since it reaches to the heavens, and makes us acquainted with the aspects and revolutions of other, and perhaps better worlds. The *utility* of this sense, in every action and pursuit of life, is obvious to all; but its contribution to our stock of happiness, can only be duly appreciated by those who have once possessed, and afterwards lost, so invaluable a blessing. Our own immortal Bard has deplored this loss in never dying strains, and with much greater feeling and pathos than his fellow sufferer—Homer. Such, indeed, is the cheering effect of the visual function on the common sensorium, that all animated beings spring, as it were, into a renovated state of existence, at each return of the light, and droop into a temporary annihilation when it is withdrawn, and the office of the visual orb suspended.

It is no wonder then, that every natural defect or accidental obstacle to the function of sight should strongly engage the attention of the medical practitioner, who, upon such occasions, is often more anxiously appealed to, than where the most dangerous disease is menacing the life of the patient. The structure and functions, indeed, of the eye, are so peculiarly beautiful and interesting, that few people of a liberal education, are unacquainted with the general outline of its anatomy, physiology, and even pathology. Who knows not the history of the blind boy restored to sight by Cheselden? Who has not joined in the eulogy pronounced upon that great genius?

If astronomy confers a kind of immortality on the man who *accidentally* discovers a single star, to what honour is that surgeon entitled, who, by *preconcerted* and scientific means, lays open the smiling face of the heavens and of the earth to the astonished view of congenital blindness!

Opacity of the crystalline lens is one of the most common obstacles to the function of sight; and the chief object of the author of the work under review, appears to be that of instituting a comparison between the different methods which have been employed for its removal, and to recommend a series of operations originating with himself. The historical part of the work embraces a sphere of patient and indefatigable research, of which we cannot possibly communicate any idea, within the limits of an analysis; we must therefore confine ourselves, almost exclusively, to the *result*, or practical inferences, deduced from comparison, meditation, invention, and experience.

Chap. I. After pourtraying the erroneous opinions of the ancients relative to cataract, and while describing the different kinds of the *capsular* form, Sir William takes occasion to combat, and apparently with success, the exploded opinion of Maître-Jan, revived by Mr. Travers, respecting the *posterior* opacity of the crystalline lens being seated in the proper tunic of the vitreous humour. The weight of evidence appears to preponderate greatly in favour of the *capsula propria* of the crystalline being the seat of the affection.

Although inflammation of the capsule may produce *capsular*, Sir W. thinks, that *lenticular* cataract is not produced in this way, but rather by a defect of vitality, or the action of the aqueous humour on the lens, when the capsule is burst or gives way.

The *adherent* cataract is produced by inflammation of the iris, and capsule of the crystalline lens; in which case, coagulable lymph is poured out from the posterior part of the iris, agglutinating that portion of the membrane to the anterior part of the capsule.

"This species of cataract is known by a fixed contraction of the dimensions of the pupil, and an irregularity of form, occasioned by the adhesions, which prevent it either from dilating or contracting." P. 31.

In what is termed the *elastic* cataract, which is usually congenital, the capsule is commonly dense and thickened. The *elasticity*, however, Sir W. affirms, is in the vitreous humour, occasioning the lens to return with a spring to its former seat, as soon as the needle which depressed it is withdrawn. In the *tremulous* cataract, an undulatory motion is produced in the iris and lens, from the slightest movement of the eye. This, Sir W. considers to be owing to the disorganization of the vitreous tunic, whereby that support to the lens is lost which is afforded by those processes of the membrane that pass over, and are united to, the anterior circumference of the crystalline capsule.

Our author informs us, that the first symptom of approaching cataract is marked by a slight obscurity of vision, as if the patient were looking through a cloud, light smoke, or dirty glass, accompanied frequently with muscæ volitantes. In this stage, there is no perceptible opacity of the lens or its capsules, and consequently it is very difficult to distinguish between cataract and gutta serena at such period. Shortly afterwards, the pupil loses its beautiful jet black colour, and assumes a turbid appearance, which increases gradually till the lens becomes entirely opake, constituting mature or ripe cataract.

During the advance of this process, unless where there is a complication of some other disease, the functions of the iris are preserved, and there is no pain or other symptom to alarm the patient. The disease generally begins in one eye; and when considerable progress is made, the other becomes similarly affected. The usual period which elapses, from the first commencement of obscure vision, until the cataract is completely formed, is between one and three years; sometimes, however, the progress is very rapid, at others very slow.

"Richter mentions the case of a forester, who, labouring under gout, had his feet exposed to a great degree of cold during the night: the gout suddenly retroceded in consequence, and he was entirely deprived of his sight the same night. He saw him next morning, and found a complete pearly-coloured cataract." P. 37.

But whatever may be the cause or duration of the affection, still, if the eye have sustained no serious organic injury, and the functions of the retina are not permanently deranged, our author hesitates not to advise an operation. Sir William here brings forward numerous instances from his own practice, and that of others, illustrating the *hereditary* tendency to cataract. When *syphilis* is the cause, which it often is, our author is of opinion that, in such cases, "the opacity is always at first seated in the *capsule* (though it may afterwards sometimes extend to the *lens*) and generally arises from an inflammation of that membrane, and not from coagulable lymph having been effused from the inflamed vessels of the iris, and deposited on its anterior surface." P. 42.

Of scrofula, as a cause of cataract, Sir W. entertains doubts, but states that violent mental emotions—continued exposure of the eyes to a strong light and heat, are among the occasional causes, especially the latter. As to internal or external remedies, experience, he thinks, has sufficiently shewn their inutility, not so say their injurious consequences to the patient's constitution, particularly when employed immediately before an operation, as the system is thereby rendered preternaturally irritable, and the eye, of course, more disposed to irritative inflammation. It is to be understood, however, that these observations are only applied to cataract occurring spontaneously. When resulting from accident, from syphilis, or other disease, admitting of remedial relief, then the cataract, especially the capsular form, may probably be relieved also. In capsular opacity from syphilis, Sir William has "very often succeeded when the opacity was so dense, as to entirely preclude the rays of light from the retina, during the incipient stage of the inflammation." P. 55.

Weakly and irritable patients, our author thinks with Scarpa, should be prepared for the operation, for some time, by the administration of tonics and nourishing food; and sould there be any other disease of the organ, or of the constitution, it should be removed by appropriate means. Sir William sometimes recommends the occasional application of a weak solution of belladonna to the eye, for the purpose of dilating the pupil, and admitting a few rays of light through the edges of the cataract, by which means the retina receives a small share of its natural stimulus, and the patient's comfort is increased.

The contra-indications of an operation should be examined into, before we proceed to that step; for instance, an extensive opacity of the cornea covering the whole of the pupil, staphyloma, dropsy of the eye, a morbid sensibility or total insensibility of the retina, &c. These forbid an operation. Our author here, in illustration of the fact that, the best oculists occasionally confound certain states of insensibility of the retina, from long continued exclusion of its proper stimulus, light, with gutta serena, states several cases where he has succeeded, after they had long been considered incurable, and abandoned by others. The state of the pupil, our author thinks, does not afford, in these cases, any sure diagnostic mark to guide us. He therefore makes it a rule to operate, whether the cataract be complicated with adhesions to the iris or not, "if the patient is capable of perceiving light from darkness, and the motion of an opake body between his eye and the light." P. 64.

### Chap. II. *History and Nature of Depression.*

The operations for cataract consist either in removing the opake body from the axis of vision, by placing it at the bottom of the eye, which is called couching or depression—extracting it altogether, or effecting its removal by the absorbents, after producing its solution in the aqueous or vitreous humour, termed by our author, the "absorbent practice;" though, strictly speaking, the object is *absorption* in the operation of depression; but that object is not always attained. Here Sir William enters into a calm, and we conceive, a very liberal examination of the different methods proposed for executing each of these operations, pointing out freely, but fairly enough, the advantages or disadvantages which may respectively attend them, before bringing forward his own improvements and new modes of operating in extraction.

Couching is coeval with the earliest records of medical history. Celsus, and almost all succeeding writers describe it; and in the East, it has been practised time immemorial. From the great liability, however, of the cataract to rise again, the operation of depression fell into general discredit, till Pott, Scarpa, and Hey revived, to a certain degree, the credit of the operation. Here Sir William Adams brings forward an extract from Mr. Pott's works, completely proving that this eminent surgeon *first* ascertained, by dissection, the power of the vitreous humour to dissolve a cataract when depressed; and he was also the first person who pointed out that the fragments, when placed in the anterior chamber, became dissolved and absorbed, without occasioning mischief to the eye.* Professor Scarpa claims this discovery for himself, but happens to be a few years too late.

In the operation of couching, the needle, whatever its form, is introduced through the sclerotic and other coats of the eye, a small distance behind the iris, and a little below its central diameter, till its point reach the centre of the cataract, when the latter is to be detached from its ciliary connexion, and depressed towards the bottom of the eye, below the axis of vision, or on one side of the pupil. After the cataract is retained in its new situation, for a short time, by the point of the needle, the instrument is withdrawn, the operation is concluded, and the patient's sight is restored.

So easy an operation, both for patient and practitioner, were there no ultimate results of a disappointing nature, would be universally adopted, especially as the solvent powers of the aqueous and vitreous humours are now well known. The difficulties and dangers too, of extraction, enhance the good opinion entertained of couching.

As Professor Scarpa is the great champion of depression, Sir William Adams here enters into a very full and critical examination of his opinions and practices. We shall quote the following passage, exhibiting the groundwork of our author's objections to Professor Scarpa's tenets.

"In the *first* place, there is no kind of cataract, the nucleus of which is capable of being divided, that is not more speedily and certainly removed by absorption, when placed in the anterior chamber, than when depressed in the vitreous humour. *Secondly*, when the vitreous humour is in a healthy condition, every depressed cataract is liable, at any time before its dissolution, either to rise, and resume its former situation, or to fall into the anterior chamber of the eye, where, from being undivided, it may occasion great pain and suffering to the patient, and even hazard the total destruction of the organ. *Thirdly*, when a hard and solid cataract, whether its capsule be lacerated or not, is depressed into a disorganized

* *See* Pott's Works, Vol. iii. p. 157.

vitreous humour, unless the nucleus be divided, it will remain so long undissolved, as by its weight and pressure on the retina, frequently to cause gutta serena; or, by its rolling about, from the motion of the head, and the continual friction on the retina or iris, to occasion such severe pains, and inflammation, as to defeat the purpose of the operation, by producing a closure of the pupil, or a suppuration, and sinking of the eye." P. 81.

For the long chain of facts and reasonings, by which these objections are supported, and we should be inclined to think, pretty fully proved, we must refer to the work itself, from page 81 to page 115, the conclusion of the second chapter.

### Chap. III. *Sect.* 1. *History and Nature of Extraction.*

Although it has been generally supposed, that St. Yves and John Petit, in 1707-8, were the first who extracted the cataract, Sir William Adams has brought forward unquestionable evidence from Pliny, Rhasis, Avicenna, &c. that the ancients practised this operation. Dr. Scott informs our author, also, that the Indians have long been acquainted with it. It was not, however, till the year 1752, that extraction was introduced, with brilliant success, by Daviel; but his instruments were too complicated; and to Wenzel and Richter is due the present mode of operation. This mode, as described by Wathen, every surgeon knows to be section of the cornea, and capsules of the lens, with gentle pressure on the eye-ball, till the cataract starts from its bed in the vitreous humour, and passes out through the pupil and external wound, on the cheek. Although St. Yves, in 1707, extracted a cataract which had *accidentally* fallen into the anterior chamber of the eye, it was not till the year 1812, that Sir William Adams adopted the practice of first placing the opake lens, by means of a needle, in the said chamber, and afterwards extracting it through an opening in the cornea. In January, 1813, our author states his having performed the same operation several times on the Greenwich pensioners, in presence of professional gentlemen. In 1814, Mr. Travers published on the same operation, affirming, in a note, that he had performed it twelve months before. The claim to *priority*, therefore, is disputed between our author and Mr. Travers; and on this and every other occasion of the kind, we simply refer our readers to the writings of the claimants themselves, if they are anxious to decide on the question in dispute; for, to use Sir William Adams's own words—

"The public probably are not very much interested in deter mining to which individual the *priority* of invention should be allotted. The fact most important for them to settle is, under whose hands the operation has been most successfully employed." P. 128.

The operation performed by Sir William Adams is adapted particularly to those cases where the nucleus of a cataract is too hard to admit of immediate division.

The following is a concise detail of the *methodus operandi.*

A weak solution of belladonna is applied *over-night,* in order that the pupil may quickly recover its natural size after the lens is pushed into the anterior chamber. The two-edged needle is first to be introduced through the sclerotica, a line behind the iris, with its flat surface parallel to that membrane. Its point is then to be directed through the posterior chamber, on a line with the transverse diameter of the opake lens, when its edge should be turned backwards, and a complete division of the capsule and lens attempted in the manner hereafter to be described. But if the lens be too hard to admit of immediate division, the point of the needle should be withdrawn a little, and then carried something below the line of the transverse diameter of the cataract, when, upon making pressure with its flat surface against the latter body, it becomes dislocated, and the upper part tilts forward through the pupil into the anterior chamber; after which, without any difficulty, it may be entirely carried through the pupil, and with its posterior part turned forwards. This effected, the operator, with the point of the needle, (taking care not to wound the iris) should lacerate, or cut in pieces, the remaining part of the capsule, throughout the whole extent of the circumference of the dilated pupil, by which means secondary cataract is certainly avoided. The needle is then to be withdrawn. So far, the patient is sitting in his chair; he is now to be placed on his back, with the head somewhat raised. The operator then makes an opening in the temporal margin of the cornea, with a lancet or double-edged extracting knife. This opening is enlarged, both upwards and downwards, with a small curved knife, until it is made sufficiently large to admit of the free passage of the lens. Through this aperture a small hook is introduced, with its flat surface between the anterior part of the iris, and the posterior part of the lens, which should be carried to the centre of the pupil; the curved point is then turned forwards, and the cataract laid firm hold of and extracted. By this plan the lens is withdrawn, without any pressure being made on the eye, and through an opening much smaller than is required in the usual operation of extraction. P. 141.

Our author justly remarks, that the various species, varieties, and combinations of this disease, can never be expected to bend to any *one* operation. The *modus operandi,* in fact, must vary with the varieties of the complaint. The leading principle of his practice is, that *solution and absorption* of lenticular cataract should *always,* if possible, and compatible with safety, be preferred. This is always practicable when the consistence of the cataract admits of free division; in which case, our author places a part, or the whole of the fragments in the anterior chamber, where they become absorbed, in the space of a few weeks, without pain or inconvenience. Where, on the contrary, the nucleus will *not* admit of this division, Sir William now extracts it at once. The advantage of this operation consists in being able to ascertain with the needle, whether the cataract admits of division or not, which is not possible in the usual mode of operating.

Chap. III. *Sect.* 2. In this Section, Sir William Adams presents a view of the different cases to which the usual operation of extraction is ill adapted, or wholly inapplicable, with descriptions of other and more appropriate methods. Extraction is scarcely practicable, for instance, in children, or where the cataract is congenital, from the difficulty of keeping the eye steady in either case, the use of the speculum being inadmissible in *extraction.* But, by this instrument, the most complete control is kept over the motions of the eye-ball, and from the soft state of the nucleus of the lens, it can always be freely divided, and the fragments easily placed in the anterior chamber of the eye, for absorption.

*Capsular* cataract is very difficult of extraction; and it is precarious in its result, in consequence of the necessity of passing the forceps or hook through the pupil, and of making so large an opening in the cornea. There is still more danger where the capsule, as is often the case, adheres to the iris.

Sir William affirms, that the dangers necessarily attendant on the operation of extraction, in the *capsular, adherent capsular, and fluid cataract,* are wholly obviated by his modes of practice. The instrument he employs, in these cases, is a needle with a very slight degree of curvature at its point, with which he ruptures the capsule very freely, when it will admit of it, until the pupil is perfectly clear throughout its whole extent. But when the capsule is so much thickened, that this laceration cannot be effected, he proceeds to detach it completely from the ciliary processes, except at one point. It should then be repeatedly pressed with the point of the needle, until the capsule can no longer be seen within the area of the pupil. This point of attachment serves to prevent the capsule from floating in the axis of vision, an evil which our author has frequently seen, when the capsule was wholly detached.

In *fluid cataract,* which is generally accompanied with an opake capsule, our author proceeds in a similar manner, preferring, however, in this case, the use of the double-edged to that of the curved needle. After the opake fluid is let out of its capsule, and is diffused in the aqueous humour, the point of the instrument should be somewhat withdrawn, and the capsule treated as described for the removal of capsular cataract, by which means the pupil becomes frequently cleared at the first operation.

When there is capsular opacity, with transparent lens, which is generally the result of inflammation, and complicated with adhesion to the iris, the difficulty and danger of extraction are greatly increased ; and here Sir William affirms, that he finds the operation with the needle both safe, easy, and effectual. The usual mode of extraction is very inapplicable to the adherent or concreted cataract, from the injury the iris sustains by the introduction of instruments through the pupil, after the section of the cornea has been made, and by the forcible separation of the iris from the capsule, and the pressure which is necessary to be made on the globe of the eye, in order to force the lens through the contracted undilatable pupil, together with the usual discharge of vitreous humour.

However extensive the adhesions of the capsule to the iris may be, if the pupil be not very much contracted, Sir W. cuts up the capsule and lens (when soft) as much as possible, before he entirely separates the adhesions, and then pushes a part, or the whole of the fragments, through the pupil into the anterior chamber for solution and absorption. Should, however, the cataract be too hard for this division, the operator must, at first, with the iris scalpel, divide the iris to three fourths the extent of its transverse diameter, and then with the point of the same instrument, liberate the adhesions, and push the undivided lens enveloped in its capsule, through the newly-formed aperture into the anterior chamber, when extraction is to be immediately performed. If the pupil remain too much contracted for the passage of light, he at once divides the iris, and forms an artificial pupil.

Extraction, in the common way, is very inapplicable to the *elastic cataract,* previously alluded to; whereas the firmness of the vitreous humour affords an admirable counter-resistance to the pressure occasioned during the central division of that opake body. In no instance of this kind has our author failed.

We must pass over Sir William's observations on the inapplicability of the common mode of extraction to flat cornea and convex iris, small sunken eye, spasms of the ocular muscles, large pupil with small lens, large lens with small pupil, cystic or hydatid cataract, &c. &c. referring our readers for more specific information to the work itself. The third Section of Chapter III. must also remain untouched, in which the author enumerates the dangers of extraction in the common mode, even where that mode is most applicable, and the "readiness with which they may be avoided by the practice of the author's operations." In this Section, the professed oculist will find a great mass of interesting and important matter.

In the fourth Chapter, the author introduces an analysis of his own operations, with a candid avowal of the dangers to which they also are liable. The following extract shows Sir William Adams's operation for solid cataracts in children and adults.

"Having secured the eye, by making gentle pressure with the concave speculum, under the upper eye-lid, I pass the two-edged needle through the sclerotic coat, about a line behind the iris, with the flat surface parallel to that membrane; it is then carried cautiously through the posterior chamber, without, in the slightest degree, interfering with the cataract or its capsule. When the point has reached the temporal margin of the pupil, I direct it into the anterior chamber, and carry it on as far as the nasal margin of the pupil, in a line with the transverse diameter of the crystalline lens. I then turn the edge backwards, and with one stroke of the instrument, cut in halves both the capsule and cataract. By repeated cuts in different directions, the opake lens and its capsule are divided in many pieces, and at the same time I take particular care to detach as much of the capsule as possible from its ciliary connexion. As soon as this is accomplished, I turn the instrument in the same direction as when it entered the eye, and, with its flat surface, bring forward into the anterior chamber, as many of the fragments as I am able. By these means, the upper part of the pupil is frequently left perfectly free from opacity. By cutting in pieces the capsule and lens at the same time, not only is capsular cataract generally prevented, but the capsule is also much more easily divided into minute portions, than when its contents have been previously removed." P. 256.

The needle which our author employs is eight-tenths of an inch long, the thirtieth part of an inch broad, and has a slight degree of convexity through its whole blade, in order to give it sufficient strength to penetrate the coats of the eye without bending; it is spear-pointed, with both edges made as sharp as possible, to the extent of four-tenths of an inch. Above the cutting part it gradually thickens, so as to prevent the escape of the vitreous humour.

Our closing limits will not permit us to follow Sir William Adams through the long chain of minute descriptions of the various operations connected with cataract, together with the series of critiques on the modes of operating followed by others. These must be perused with care by those surgeons who, themselves, operate on the eye; whereas, in this analysis, we have dwelt most on those *general points of ophthalmologic knowledge* with which every Physician, Surgeon, and general Practitioner, ought to be acquainted.

Towards the close of the volume, Sir William Adams has presented a number of documents, evincing the comparative success of the different operations for cataract. From these, it would appear that the operation of extraction, in the hands of Daviel, was successful in little more than half the patients operated on, and that this has been nearly the ratio of success attending depression and extraction, even in the hands of the most eminent oculists of our own day. In the Hotel Dieu of Paris, about two out of five patients recover their sight after the operation for cataract. Our author, on the contrary, states, that while at Exeter, he operated on forty persons in succession, without experiencing a single failure. We sincerely wish that the future experience of others may confirm this unexampled success; but we know enough of human nature, not to be convinced that every man who invents a new plan, sees it through too favourable a medium, especially at first.

Of the *matter* of the work before us, we hope we have enabled our readers to judge for themselves, though we cannot but think that the author would have rendered a greater service, both to himself and the profession, if he had compressed it into narrower bounds, by entering less deeply into the historical parts. The *manner* of the work we cannot so much praise, since a fastidious critic might, with some justice, object to several passages.

Into the papers and documents at the end of the volume, we shall not enter. Both sides of the contest between our author and his opponents are fairly before the public, and God forbid that we should arrogate to ourselves the office of *judge* or umpire, when the *jury* of the profession is appealed to. In cases of this nature, we conceive it our bounden duty, either to lay a fair statement of both sides of the question before the public, without comment, or to remain entirely silent. As the *former* is inconsistent with the plan and purport of our Journal, the alternative we must adopt.

